# Staufen purification: challenges and opportunities. Protocols and troubleshooting^✰^

**DOI:** 10.1016/j.mex.2026.103902

**Published:** 2026-04-08

**Authors:** Huma Shakoor, Andrea Tripepi

**Affiliations:** a Central European Institute of Technology, Masaryk University, Kamenice 753/5 62500 Brno, Czech Republic; b National Center of Biomolecular Research, Kamenice 753/5 62500 Brno, Czech Republic

**Keywords:** Protein purification, Aggregation, Degradation, FPLC, EDTA, Staufen

## Abstract

Protein purification is required for many experimental assays in molecular biology. However, this is a laborious procedure that can be challenging and prone to several problems (degradation, aggregation, contamination etc.). These issues can jeopardize the quality of the samples and the reliability of the research tests. This article describes four protocols that can be used for the purification of human Staufen1 (and several mutants), an important protein capable of binding RNA and inducing a variety of phenomena crucial for cell biology, including Staufen-mediated mRNA decay (SMD). SMD dysregulation is reported to be involved in tumorigenesis, adipogenesis, neurodegeneration, and cell cycle regulation. The data presented here show that EDTA reduces protein degradation. These protocols can minimize Staufen degradation and aggregation; therefore, they have proven to be efficient and reliable. This article also provides a table of potential problems and their corresponding solutions. Moreover, this work shows that the removal of a Staufen domain (Staufen-Swapping Motif, SSM) highly increases the degradation of this protein. This suggests that SSM plays a role in Staufen integrity.

Fast, reliable purification protocols, ideal for Staufen and other water-soluble proteins

Staufen purification troubleshooting

SSM deletion increases Staufen degradation

## Graphical abstract

**Figure:**
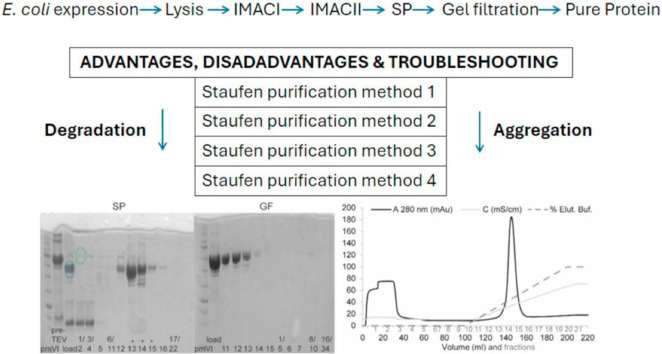

## Specifications table

*This table provides general information on our method.*

**Table:** 

Subject area	Biochemistry, Genetics and Molecular Biology
More specific subject area	*Protein production and purification*
Name of your method	*Staufen purification method 1, 2, 3, 4*
Name and reference of original method	*This is an original method*
Resource availability	*The resources are mentioned in the material section*

## Background

Protein purification is required in many experiments in molecular biology. However, it is necessary to consider that protein purification requires laborious procedures and poses significant challenges [1,2 ,3]. The chemical, physical, and biological features of proteins can vary greatly from one to another. Such intrinsic variety requires sample-dependent optimization of the purification procedures. There are several issues that can jeopardize the purification process. For example, protein degradation and/or sedimentation can severely damage the quality of the samples; for this reason, avoiding these problems is crucial for obtaining reliable results [4]. To prepare a proper purification protocol, it is necessary to consider various parameters that can vary significantly from protein to protein. For example, it is necessary to carefully evaluate protein pI, water solubility, molecular weight, cysteine content, amino acid composition, stability, and sedimentation [[5], [6], [7], [8]].

Many proteins are used in molecular biology, one of these proteins is receiving a growing interest in the scientific community: Staufen. This protein plays vital roles in cell biology. In mammals, there are two forms of this protein: Staufen 1 and Staufen 2, which are expressed throughout the body and in the Central Nervous System, respectively. Staufen1 is known to be involved primarily in mRNA transport and degradation (Staufen-mediated mRNA decay-SMD), which, in turn, is deeply involved in tumorigenesis, adipogenesis, neurodegeneration, cell cycle regulation, and many other phenomena [[9], [10], [11], [12]].

**Fig. 1:**
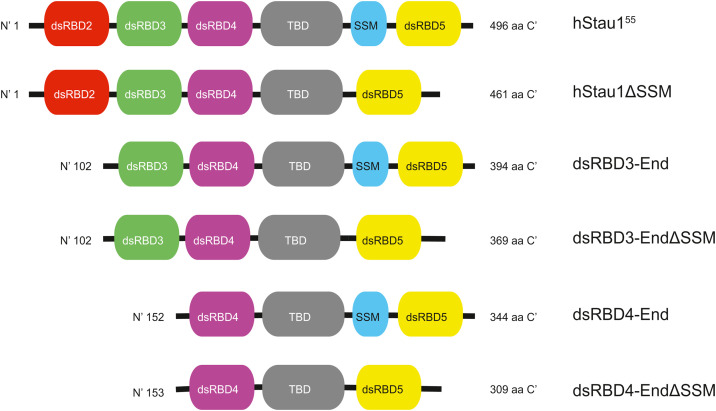
the schematic structure and the domains of the proteins hStau1, hStau1ΔSSM, dsRBD3-End, dsRBD3-EndΔSSM, dsRBD4-End, dsRBD4-EndΔSSM.

Staufen1 can recognize dsRNA secondary structures by its dsRBD3 and 4. This protein also possesses a Tubulin Binding Domain (TBD) that is required for tubulin binding and for RNA transport [13]. This protein also contains a Staufen Swapping Motif (SSM), which is characterized by two α-helices. This domain is reported to be required for Staufen dimerization [14]. Overall, Staufen1 structure is characterized by folding domains spaced by disordered linkers and can display a variety of structures (SASBDB structure: SASDW9) (Fig. 1 and Sup. Fig. 1).

It is interesting to note that Staufen1 degradation is reported to be connected to inflammation and cancer. It is also reported that dsRBD2 is targeted by proteases [15].

In a report [14] Staufen pellets were suspended in ∼40 ml of Buffer A (1 M NaCl, 25 mM Tris–HCl pH 8) to which was added 55 μl of 0.93 M dithiothreitol (DTT), 500 μl of 100 mM PMSF, 50 μl of 0.5 M EDTA pH 8, 500 μl of 80 mg ml−1 lysozyme, and a protease inhibitor tablet (Roche). They run the sample in GSTrap™ HP column and elution was performed in their GF buffer (100 mM NaCl, 10 mM Tris–HCl pH 8, 1.3 mM DTT with glutathione (0.3 g in 100 ml buffer). In another study, DTT was also used in Staufen dsRBD3–4 purification [16]. It is necessary to consider that DTT can negatively alter antibody binding and tagging with fluorescent dyes [17,18], therefore, it can jeopardize ELISA, Western Blot, fluorescence anisotropy or other similar assays and tests.

In another report [19], hStau1ΔdsRBD2 (which corresponds to our dsRBD3-End), cell pellets were suspended in 25 mM HEPES pH 7.5, 1 M GndCl, 20 mM imidazole, 1 % Triton X-100. Nickel chromatography was performed in HisTrap FF columns (GE Healthcare) equilibrated in washing buffer (25 mM HEPES pH 7.5, 1 M GndCl, 20 mM imidazole), with increasing imidazole concentration in elution until 1 M. After this step, gel filtration was performed in 25 mM HEPES pH 7.5, 100 mM KCl, 10 mM MgCl2, 200 mM l-Arg HCl). Hepes was also used by other researchers for Staufen dsRBD3–4 protein purification [[13], [16]]. HEPES is used to stabilize buffer pH but, in our experience and in another report [20], can negatively impact measurements of protein concentration made by spectrophotometer. Moreover, none of these protocols use EDTA or other compounds to reduce protein degradation.

Considering all these facts, proper Staufen purification is crucial in many research areas. Some of the proteins described here were difficult to purify and prone to degradation and/or aggregation. Here are described reliable methods to prevent, or at least reduce, protein degradation and aggregation in Staufen proteins with and without SSM. In the troubleshooting section, you can find tips to minimize these issues.

## Method details

### Materials

• Antarctic phosphatase (NEB, USA, Cat. Number: M2200)
• BL21-CodonPlus (DE3) -RIL E. coli cells (Agilent, USA, Cat. Number: 230245 RUO)
• BME (Sigma-Aldrich, Germany. Cat. Number: 102448170)
• cOmplete EDTA-free protease inhibitor (Sigma-Aldrich, USA, Cat. Number: S8820)
• Dh5α E. coli cells (Thermofisher, USA, Cat. Number: EC0111)
• EDTA (PanReac AppliChem, Germany. Cat. Number: A1103,1000)
• Glycerol (VWR Chemicals, Belgium. Cat. Number: 24388.295)
• Imidazole (PanReac AppliChem, Germany. Cat. Number: 162536.1211)
• NaCl (Penta Chemicals Unlimited, Czech Republic. Cat. Number: Q09101)
• NdeI (Takara, Japan, Cat. Number: 1061A)
• Plasmid p28a (Addgene, Cat. Number: 141289)
• Phusion Polymerase High Fidelity (NEB, USA, Cat. Number: F30S)
• Protein marker VI prestained (VWR, USA, Cat. Number: 35748464)
• T4 ligase (Takara, Japan, Cat. Number: 2011B)
• TEV Protease (Produced in our lab)
• Tris (PanReac AppliChem, Germany. Cat. Number: A1379.1000)
• Tryptone (Merck, USA, Cat. Number: 110694)
• Water Chromasolv® Plus, for HPLC (Sigma-Aldrich, Germany: Cat. Number: 7732-18-5)
• XhoI (Takara, Japan, Cat. Number: 1094A)
• Yeast Extract (Merck, USA, Catalogue Number: Y1625)

### Equipments

• AKTA PrimePlus (Cytiva, Sweden, Cat. Number: 11,001,313)
• EmulsiFlex C3 cell cracker (Avestin, UK, Cat. Number: 3346,020)
• HiTrap™ IMAC HP 5 ml column (Cytiva, Sweden, Cat. Number: 17,092,005)
• HiTrap™ SP HP cation exchange column (Cytiva, Sweden, Cat. Number: 17,115,201)
• Nanodrop (Thermofisher, USA, Cat. Number: ND2000)
• QIAquick Gel Extraction Kit (QIAgen, Germany, Cat. Number: 28,704)
• Spectra/Por dialysis membrane (Spectrum Laboratories, USA, Cat. Number 132680T)
• Superdex™ 200 pg SEC column (GE, USA, Cat. Number: 28,989,335)
• Superdex™ 75 pg SEC column (GE, USA, Cat. Number: 28,989,333)
• Vivaspin 20 Centrifugal concentrator (Sartorius, Germany, Cat. Number: VS2001)

### Buffers

• His-A buffer (1 M NaCl, 50 mM Tris–HCl pH 8, 30 mM imidazole, 10 % glycerol (w/v), and 3.8 mM β-mercaptoethanol)
• His-B buffer (0.5 M NaCl, 50 mM Tris–HCl pH 8, 600 mM Imidazole, 10 % glycerol (w/v), and 3.8 mM β-mercaptoethanol)
• TEV buffer (0.5 M NaCl, 50 mM Tris–HCl, pH 8, 10 % glycerol (w/v), and 3.8 mM β-mercaptoethanol)
• Buffer A (0.1 M NaCl, 50 mM Tris pH 8, 3.8 mM β-mercaptoethanol, and 0.25 mM EDTA)
• Buffer B (1 M NaCl, 50 mM Tris pH 8, 3.8 mM β-mercaptoethanol, and 0.25 mM EDTA)
• Buffer A* (300 mM NaCl, 50 mM Tris pH 7, 3.8 mM β-mercaptoethanol, and 0.5 mM EDTA)
• Buffer B* (1 M NaCl, 50 mM Tris pH 7, 0.5 mM, 3.8 mM β-mercaptoethanol, and 0.5 mM EDTA)
• GF buffer (150 mM NaCl, 50 mM Tris pH 8, and 3.8 mM β-mercaptoethanol)
• GF* buffer (300 mM NaCl, 50 mM Tris-Hcl pH 7, and 3.8 mM β-mercaptoethanol)

### Gene cloning

Gene cloning was performed by producing specific amplicons by polymerase chain reaction (PCR) using appropriate primers (Table 1). The sequences of the primers are listed in Table 1. These amplicons were subcloned into the pET28a vector with kanamycin resistance [21] with a TEV instead of the original thrombin cleavage site and a N-terminal His_6_-Strep-His_6_-tag or His_6_-lipo-tag (Sup. Fig. 1) using the NdeI and XhoI restriction sites as previously described [13] (i.e., to create a hStau1ΔSSM, a plasmid bearing full Staufen1 was used) for DNA amplification. To delete SSM, two amplification steps were performed using two different primer pairs (Table 1) based on the DNA domain deletion protocol [22]. Amplicons were run in 1 % agarose gel. Extraction was performed by QIAquick Gel Extraction Kit (QIAgen, Germany) according to the protocol recommended by QIAgen, and the concentration of amplicons was measured by Nanodrop (Thermofisher, USA). Digestion of the expression vector was performed by XhoI and NdeI endonucleases (Takara, USA). This step was followed by Antarctic Phosphatase treatment (NEB, USA). Ligation of cut amplicons with pET28a vector was performed by T4 Ligase (Takara, Japan) in a 3:1 molar ratio at 16°C overnight. The cloned plasmids were used to transform Dh5α and BL21-CodonPlus (DE3)-RIL *E. coli* cells. The transformed bacteria were plated onto a LB plate containing 30 mg/L kanamycin. DH5α cells were used to store plasmids, and BL21-CodonPlus (DE3)-RIL cells were used to produce proteins. *E. coli* cells were stored in 30 % glycerol at −80°C. Patent pending for proteins with deleted or modified SSM, patent application 102026000009622 submitted to the Italian Ministry of Entreprises and Made in Italy*.*

**Table 1:** List of primers used in our plasmid constructs.

Plasmid constructs	Forward primer	Reverse primer
hStau1	5′-ATCGGCGGCCGCAAACTTGGAAAAAAACC-3′	5′-GCTAGTTATTGCTCAGCGGTGGCAGCAGCC-3′
dsRBD3-End	5′-CCAGGGTAGCCATATGAAACCCGCACTCAAGTCAGAGGAG-3′	5′-GCTAGTTATTGCTCAGCGGTGGCAGCAGCC-3′
dsRBD4-End	5′-TCCATATGAATCCGATAGCCGACTGGCC-3′	5′-GCTAGTTATTGCTCAGCGGTGGCAGCAGCC-3′
hStau1∆SSM	Forward primer 1 5′-CGACTCACTATAGGGGAATTGTGAGCGG-3′ Forward primer 2 5′-GTAACTGCCCCCTCTGAGCAACTGGAC-3′	Reverse primer 1 5′-CTCAGAGGGGGCAGTTACCGTGGCCTTG-3′ Reverse primer 2 5′-GCTAGTTATTGCTCAGCGGTGGCAGCAGCC-3′
dsRBD3-End∆SSM	Forward primer 1 5′-CGACTCACTATAGGGGAATTGTGAGCGG-3′ Forward primer 2 5′-GTAACTGCCCCCTCTGAGCAACTGGAC-3′	Reverse primer 1 5′-GCTAGTTATTGCTCAGCGGTGGCAGCAGCC-3′ Reverse primer 2 5′-CTCAGAGGGGGCAGTTACCGTGGCCTTG-3′
dsRBD4-End∆SSM	Forward primer 1 5′-CGACTCACTATAGGGGAATTGTGAGCGG-3′ Forward primer 2 5′-GTAACTGCCCCCTCTGAGCAACTGGAC-3′	Reverse primer 1 5′-CTCAGAGGGGGCAGTTACCGTGGCCTTG-3′ Reverse primer 2 5′-GCTAGTTATTGCTCAGCGGTGGCAGCAGCC-3′

### Pellet production

Transformed *E. coli* RIL cells were inoculated into liquid LB with 30 mg/L kanamycin. The cultures producing dsRBD3-End, dsRBD3-EndΔSSM, dsRBD4-End, dsRBD4-EndΔSSM (Fig. 1), were grown at 37 °C until 0.8–1.0 OD and moved to 16 °C for 30 min. Induction was then performed by IPTG (final concentration 1 mM) at 16 °C for 16 h. For bacteria producing hStau1 and hStau1ΔSSM, induction was performed at 25 °C. After cell pelleting, the pellets were resuspended in His-A buffer, and a protease inhibitor (cOmplete EDTA-free, Sigma-Aldrich, USA) was added (0.5 tablets/liter of medium).

### General procedures

**Fig. 2:**
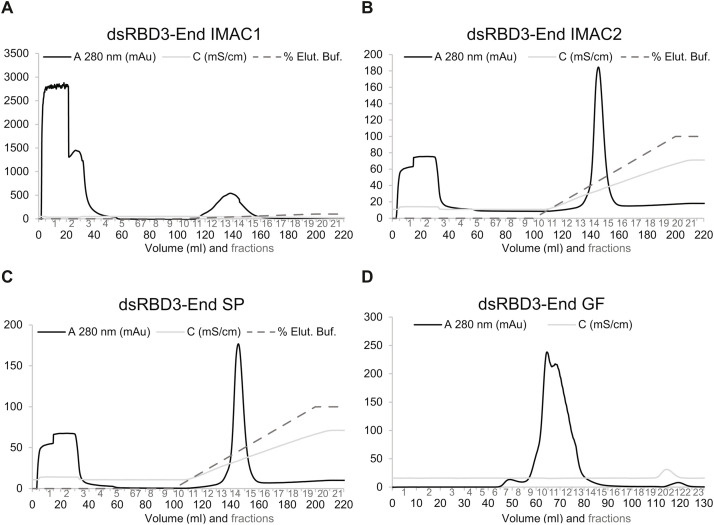
(A) dsRBD3-End IMAC I; (B) IMAC II; (C) cation exchange (SP) and (D) gel filtration (GF) chromatograms. Absorbance at 280 nm, conductibility, percentage of elution buffer data are reported. The protocol used was Staufen purification method 2.

**Fig. 3:**
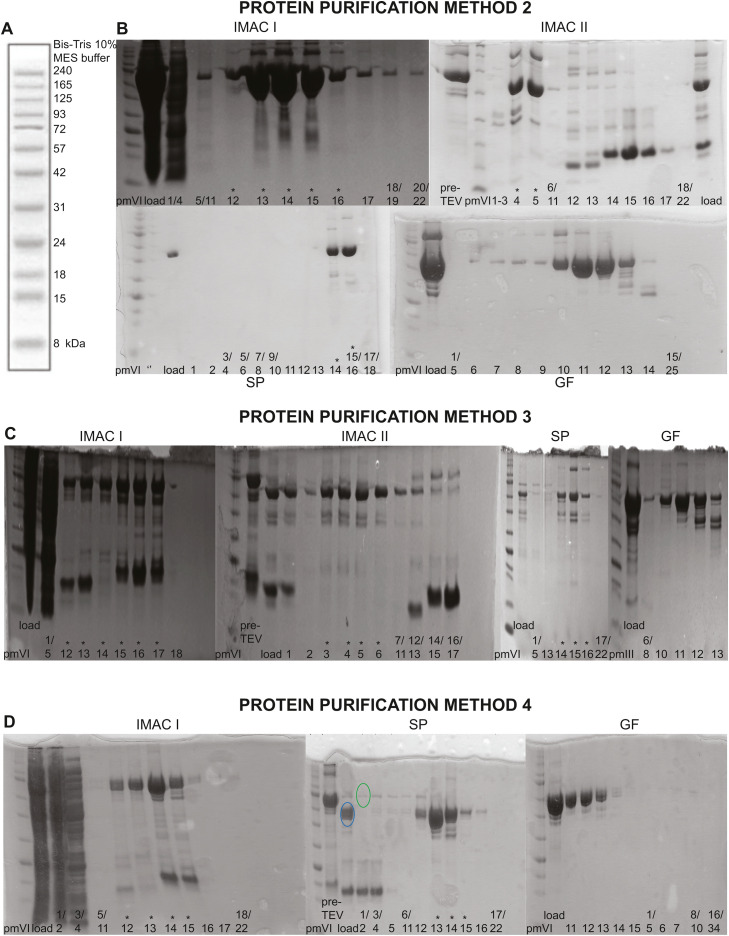
(A) pmVI marker scheme; (B) dsRBD3-End SDS pages (Staufen purification method 2); (C) hStau1 SDS pages (Staufen purification method 3); (D) hStau1ΔSSM SDS pages (Staufen purification method 4) (cut and uncut protein particles are inside blue and green circles, respectively). IMAC I, IMAC II, SP, GF steps are mentioned in the figure from above to bottom and from left to right.

The procedures here presented require FPLC (fast protein liquid chromatography) runs. FPLC fractions need to be raced into 12 % SDS page to check sample integrity. We performed all FPLCs (Fast Protein Liquid Chromatography) in Cytiva AKTA PrimePlus (Sweden) devices. We used 50 ml sample loop in all FPLC, except for gel filtration. In the latter case, we used a 5 ml sample loop. Buffer composition is reported in Table 2. Absorbance (at 280 nm), percentage of flow-through and elution buffer, pressure and conductivity data can be observed in Fig. 2. IMAC I, IMAC II, and SP flowrate was 1 ml/min in flow-through and 4 ml/min in elution. IMAC I, IMAC II, and SP flow-through buffer volume was 100 ml, elution buffer was gradually added until reaching 100 % (Fig. 2). In gel filtration there is no change in buffer composition, and flowrate was constant 1 ml/min. Examples of SDS pages are shown in Fig. 3, where the fractions taken for the following purification steps are marked with a *.

**Table 2:** List of Buffers.

Buffer Name	Buffer composition
His A buffer	1 M NaCl, 50 mM Tris–HCl pH 8, 30 mM imidazole, 10 % glycerol (w/v) and 3.8 mM β-mercaptoethanol
His B buffer	0.5 M NaCl, 50 mM Tris–HCl pH 8, 600 mM Imidazole, 10 % glycerol (w/v) and 3.8 mM β-mercaptoethanol
TEV buffer	0.5 M NaCl, 50 mM Tris–HCl pH 8, 10 % glycerol (w/v) and 3.8 mM β-mercaptoethanol
Buffer A	0.1 M NaCl, 50 mM Tris pH 8, 3.8 mM β-mercaptoethanol, 0.25 mM EDTA
Buffer B	1 M NaCl, 50 mM Tris pH 8, 3.8 mM β-mercaptoethanol, 0.25 mM EDTA
Buffer A EDTA	0.1 M NaCl, 50 mM Tris pH 8, 3.8 mM β-mercaptoethanol, 0.25 mM EDTA
Buffer B EDTA	1 M NaCl, 50 mM Tris pH 8, 3.8 mM β-mercaptoethanol, 0.25 mM EDTA
Buffer A* for hStau1 and hStau1ΔSSM	300 mM NaCl, 50 mM Tris pH 7, 3.8 mM β-mercaptoethanol, EDTA 0.5 mM
Buffer B* for hStau1 and hStau1ΔSSM	1 M NaCl, 50 mM Tris pH 7, 0.5 mM, 3.8 mM β-mercaptoethanol, 0.5 mM EDTA
GF buffer for Staufen purification method 1 and 2	150 mM NaCl, 50 mM Tris pH 8, and 3.8 mM β-mercaptoethanol
GF* buffer for Staufen purification method 3 and 4	300 mM NaCl, 50 mM Tris-Hcl pH 7, and 3.8 mM β-mercaptoethanol
FA buffer	150 mM NaCl, 50 mM Tris–HCl pH 8.0, 3.8 mM β-mercaptoethanol and 0.25 mM EDTA

To preserve protein integrity, we slowly defrost cell pellets. This step was followed by high pressure pulses (∼1500 bar) cell cracking (EmulsiFlex C3, Avestin, UK). Alternatively, ultrasound cell cracking could be used. This step was followed by cell lysate centrifugation at 39′000 g for 45 min. We run the supernatant through IMAC column in His-A buffer for flow-through (until 100 ml; Fig. 2) and in His-B buffer for elution (˃ 100 ml). Elution buffer is gradually added to the column (also called buffer B; Fig. 2). We call this step IMACI (Fig. 2).

IMAC 1 relevant fractions were taken from the IMAC eluate and moved into a dialysis bag. In all protocols (Staufen purification method 1, 2, 3), except in Staufen purification method 4, TEV cleavage was performed in 2 L TEV buffer (1 mg TEV protease was added to the chosen fractions inside the dialysis bag). TEV buffer needs to be agitated by a magnetic stirrer at room temperature (∼ 22 °C). In Staufen purification method 4 TEV cleavage was performed in buffer A*, also in this case we used 1 mg TEV protease per sample. A new FPLC run on IMAC column (IMAC II) was used to extract untagged protein in the previous buffers. The protein samples were taken from IMAC flow-through, moved to a dialysis bag and dialyzed in 2 L buffer A agitated by a magnetic stirrer. This step is required to remove TEV protease and uncut protein.

Cation exchange chromatography was performed in SP-Sepharose columns. The composition of the buffers used in this step changes according to the purification protocol and it is reported in Table 2. In this step protein particles with RNA are discarded in the flow-through and RNA-free protein particles are eluted. In the IMAC and in the ion exchange steps the volumes of flow-through and elution are the same, as well as the gradually rising concentration of elution buffer. We recommend to use only SP-eluted protein fractions for the following purification steps.

SP-eluted fractions were concentrated by 10 kDa centrifugal concentrators and inserted in a 5 ml sample loop for a size-exclusion chromatography. This step was run into Superdex™ 200 pg column. GF buffer was used in Staufen purification methods 1 and 2, GF* buffer was used for Staufen purification methods 3 and 4.

### An example of dsRBD3-End purification (Staufen purification method 2)

After cell lysis and centrifugation, the supernatant (containing dsRBD3-End protein) was first purified by His-tag affinity chromatography (IMAC I). An imidazole gradient was setup by His-B buffer (Table 2) gradual addition (Fig. 2) and dsRBD3-End was eluted. The chromatogram shows the typical result of a successful affinity chromatography experiment (Fig. 2A), where a high *E. coli* concentration of protein is visible in the flow-through fractions (Fig. 2A and 3B). The protein concentration decreases during the washing step (Fig. 3B). An absorbance peak was observed in fractions 12–16 whose molecular weight corresponds with the protein of interest with double histidine tag (Fig. 2A), therefore, the sample was eluted at higher imidazole concentrations because the protein was strongly bound to the Ni^+2^-matrix. The result shows protein of 58 kDa in lanes corresponding to lysate, flow-through and protein fractions (Fig. 3B). Selected fractions (marked with * in Fig. 3) of protein from the affinity chromatography were dialyzed against 2 L TEV buffer for TEV cleavage inside a dialysis bag.

As already mentioned, the second run (IMAC II) of affinity chromatography was performed to separate the TEV protease and the uncut protein from the cut protein. The chromatogram shows very sharp and narrow peak in fractions 14–16 that corresponds to TEV protease and our target protein that is free of His-tag eluted in the flow-through in fraction 3–5 (Fig. 2B). To explain this low Staufen absorbance it is necessary to consider that this protein does not contain tryptophane residues. Flow-through fractions were collected and dialyzed against an ion exchange buffer called buffer A. The SP chromatogram shows peaks (Fig. 2C); flow-through contains aggregated proteins or particles bound to RNA molecules (which are present in the *E. coli* cell lysate) (Fig. 3C). The protein was eluted in Buffer B. Finally, the protein was purified by gel filtration chromatography to analyze size homogeneity. The sample derived from the ion exchange elution was applied to the superdex™ 200 pg column. Protein was eluted with a flow rate of 1 ml/min. Due to the flexible nature of the proteins here described and to their multiple structures (for more information read PRO-25–0795 submission), the chromatogram shows several protein peaks at ∼60–80 ml (fractions 10–13) (Fig. 2D).

### Staufen purification method 1

This protocol does not require EDTA, and it was initially used to purify the proteins described here. Due to high degradation rate, we abandoned this method, therefore, we decided to use protein purification method 2, 3, 4. However, this method is made of the following steps:

1. Cell cracking
2. Centrifuge the lysate at 39.000 g for 40 min at 4 °C
3. Insert the supernatant into AKTA FPLC through a 0,45 µm filter
4. Run FPLC IMAC I in His A and His B buffer (Table 2) for flow through and elution, respectively. Elution buffer is gradually added until reaching 100 %, Fig. 2). Flowrate 1 ml/min in flow-through and 4 ml in elution.
5. Overnight TEV cleavage of the fractions containing the protein and dialysis in freshly prepared 2 L TEV buffer (Table 2).
6. If the sample volume is higher than the sample loop volume, concentrate the elution fractions by a 10 kDa protein concentrator. We used Vivaspin 20 concentrators (Sartorius, Germany).
7. Run IMAC II in His A and His B buffer for flow through and elution, respectively (same as step 4). Elution buffer is gradually added until reaching 100 %, Fig. 2). Flowrate 1 ml/min in flow-through and 4 ml in elution.
8. Dialysis in agitation of protein in freshly prepared unfiltered SP-Sepharose buffer A for at least 6 h.
9. Run FPLC in SP-Sepharose column in EDTA free buffer A and buffer B. Elution buffer is gradually added until reaching 100 %, Fig. 2). Flowrate 1 ml/min in flow-through and 4 ml in elution.
10. Concentrate the elution fractions to 1 ml that contain intact protein by a 10 kDa protein concentrator, we used Vivaspin 20 concentrators (Sartorius, Germany).
11. Add 15 µL SuperaseIN (Thermofisher, USA) to the sample and run it on a pre-equilibrated gel filtration Superdex™ 200 pg column (Avantor, USA) (Table 2).
12. GF fractions that contain protein characterized by lowest degradation can be used for further experiments

### Staufen purification method 2

This method is equivalent to method 1, but it requires buffer A and buffer B (Table 2) for replacing EDTA free buffer A and buffer B, respectively. We suggest this method for dsRBD3-End, dsRBD3-EndΔSSM, dsRBD4-End, dsRBD4-EndΔSSM purification.

### Staufen purification method 3

This method is equivalent to method 2, but it requires buffer A*, buffer B* (Table 2) for dialysis and for FPLC in the SP column, as well as GF buffer 2 for gel filtration. This method is suggested for hStau1 purification, in fact, the higher NaCl concentration in buffer A* in respect to buffer A (0.3 and 0.1 M, respectively), reduce the risk of protein sedimentation which is particularly evident in human Staufen1.

### Staufen purification method 4

This protocol is equivalent to Staufen purification method 3, but it requires skipping IMAC2 FPLC and dialysis in buffer A after IMAC1. The TEV cleavage was performed in buffer A*. In this protocol, TEV cleavage was performed in buffer A* at 4 °C. We used 1 mg TEV protease per sample. We suggest to use this method for hStau1ΔSSM. In our experience, TEV cleavage was also properly performed in buffer A* and, due to the pI differences, TEV protease and uncut protein (green circle in Fig. 2D) were discarded in the flow-through fractions, instead, cut protein particles (blue circle in Fig. 2D) were found in elution fractions.

### Protein degradation essay

Protein samples were run on the SDS page. Protein degradation was determined using the ImageJ program. The gel lanes were plotted. The percentage of degradation was defined as the percentage ratio between the surface area of the bands containing degraded proteins and the total area of all the bands in a single gel lane (both degraded and undegraded).

## Method validation

### Degradation essay results

**Fig. 4:**
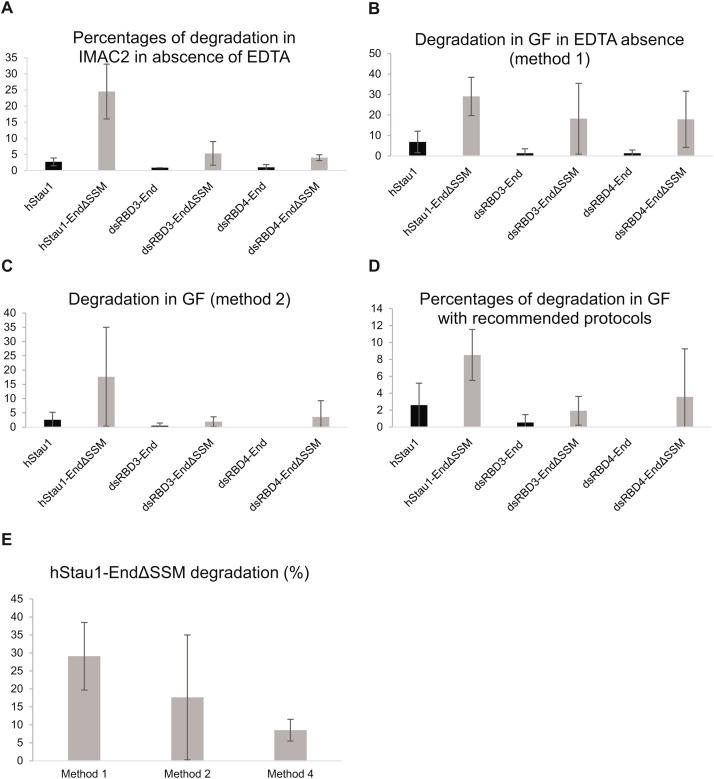
(A) percentages of degradation after 16 h in TEV buffer in hStau1, hStau1ΔSSM, dsRBD3-End, dsRBD3-EndΔSSM, dsRBD4-End, dsRBD4-EndΔSSM; (B) percentages of degradation in the cleanest GF fraction in hStau1, hStau1ΔSSM, dsRBD3-End, dsRBD3-EndΔSSM, dsRBD4-End, dsRBD4-EndΔSSM purified by Staufen purification method 1; (C) percentages of degradation in the cleanest GF fraction in hStau1, hStau1ΔSSM, dsRBD3-End, dsRBD3-EndΔSSM, dsRBD4-End, dsRBD4-EndΔSSM purified by protein purification method 2; (D) percentages of degradation in the cleanest GF fraction in hStau1, hStau1ΔSSM, dsRBD3-End, dsRBD3-EndΔSSM, dsRBD4-End, dsRBD4-EndΔSSM purified by the suggested methods; (E) percentages of degradation in the cleanest GF fraction in hStau1ΔSSM after Staufen purification method 1, 2, 4. Error bars represent standard deviations.

At the beginning, in the Staufen purification protocol (protein purification method 1), EDTA was not used, and protein degradation was a serious issue in all ΔSSM proteins (Fig. 4A and Sup. Fig. 2B, C). This phenomenon was evident after 16 h TEV cleavage in TEV buffer, especially in hStau1ΔSSM, where it was easy to see this effect in a time-dependent mode even after 3 h (Sup. Fig. 2C). At this step, the percentages of degradation were 2.7, 24.51, 0.9, 5.33, 1.01 and 4.01 % in hStau1, hStau1ΔSSM, dsRBD3-End, dsRBD3-EndΔSSM, dsRBD3-End, dsRBD3-EndΔSSM; respectively (Fig. 4A). In this protocol, the percentages of degradation were higher in the following purification steps. In fact, even in the cleanest GF fraction in the absence of EDTA (protein purification method 1) in hStau1, hStau1ΔSSM, dsRBD3-End, dsRBD3-EndΔSSM, dsRBD3-End, dsRBD3-EndΔSSM were 6.86, 29.07, 1.3, 18, 1.29, 17.9 %, respectively (Fig. 4B).

To reduce degradation, EDTA was added after IMAC II (Staufen purification method 2). This protocol highly improved the purity of the samples. In this case, the protein degradation in the cleanest GF fraction was highly reduced (*p* < 0.001) in hStau1, hStau1ΔSSM, dsRBD3-End, dsRBD3-EndΔSSM, dsRBD3-End, dsRBD3-EndΔSSM to 2.59, 17.67, 0.54, 1.92, 0, 3.57%, respectively (Fig. 4C). As expected, full length hStau1ΔSSM was again the protein characterized by the highest relative degradation rate in these conditions (Fig. 4C).

It is necessary to note that protein aggregation was a serious issue in hStau1. To prevent this problem, the pH was reduced to 7.0, and the NaCl and EDTA concentrations were increased to 0.3 M and 0.5 mM, respectively. Therefore, the dialysis before the cation exchange FPLC run was performed in buffer A* and not buffer A (protein purification method 3). Since proteases are virtually absent after the cation exchange step, EDTA was removed in the gel filtration step to prevent the issues that this compound can cause in several biochemical tests [23]. This protocol is called Staufen purification method 3.

To further reduce proteolysis in hStau1ΔSSM, the IMAC II step was skipped to minimize the time during which the protein was not protected by EDTA. In this procedure, TEV cleavage was performed in buffer A*. This protocol (Staufen purification method 4) allowed us to reach a degradation of 8.5 % in the cleanest GF fraction (Fig. 4D, E). This value is the lowest degradation rate for this protein. Considering the characteristics of this protein and its tendency to degradation, this value is acceptable for many tests.

### General remarks

EDTA is a well-known compound that chelates cations; it is commonly used to reduce protein degradation and aggregation [24]. Despite its removal being required to perform several tests [25], EDTA is often used in these applications. Our results confirm the protective effect of EDTA on protein integrity. In fact, this compound strongly reduced degradation and aggregation. This compound is very useful for minimizing proteolysis in ΔSSM and hStau1 proteins, and therefore, it is also beneficial for the purification of such biomolecules. The fact that EDTA is also a cheap and widely available molecule, make this compound an option that should be seriously taken in consideration for ΔSSM protein purification protocols. EDTA removal was performed by running the samples through a GF column in a buffer without this compound. Since EDTA and the proteins have very different molecular weights, this procedure ensures virtually complete removal of this molecule.

In our experiments, hStau1ΔSSM was the protein that had the highest tendency to degradation. This issue seems to be linked to its high disorder (submission PRO-25–0795). This protein was so prone to degradation that our purifications displayed poor quality, even when using Staufen purification method 2. The addition of 0.25 mM EDTA after the second FPLC on the IMAC column was insufficient to minimize degradation in this protein. To ensure an acceptable sample quality for many applications, we used protein purification method 4. In this protocol, it is necessary to skip the second FPLC step using the IMAC column and perform TEV cleavage in buffer A* (containing EDTA 0.5 mM) and FPLC in SP cation exchange column. This method minimized protein degradation and ensured the quality of samples that, in our opinion, was acceptable. Without this skipping, the quality of full-length hStau1ΔSSM samples was too low to obtain reliable data. Thus, the standard deviation (Fig. 2) demonstrates that, without skipping IMAC2 FPLC, the degradation rate was too unpredictable, and the chances of unsuccessful purification were high. Obviously, speed and cleaning are also essential, especially with this protein. To minimize problems of protein aggregation and degradation, the time gap between cell lysis and FPLC in SP column should be shorter than 24 h.

Despite all the efforts, sample degradation or contamination can occur. For this reason, gel filtration needs to be performed. The first GF fractions that contain protein samples often do not display signs of visible degradation, but they may contain oligomers or aggregates. On the other hand, the last fractions contain the sample, which may be characterized by high degrees of degradation. This trend was also visible in the proteins described here. Ideally, FPLC run should be set to yield an adequate number of fractions that contain pure and undegraded (or at least, with the lowest degree of degradation) samples for further analysis.

As already mentioned, Staufen purification can be troublesome, and many problems can occur. In the troubleshooting section, a list of common Staufen purification problems is provided, along with solutions that can be adopted to avoid or minimize these issues.

According to these results, the presence of SSM increases Staufen-1 stability and provides a degree of protection from proteases. The pronounced degradation observed when this domain is deleted suggests that the roles of SSM are much more varied and diverse than previously believed.

In general, purification of Staufen1-derived proteins can be challenging. It can also prove to be time- and material-consuming. The chances of purifying samples of poor quality and/or quantity can be high if proper protocols are not followed. Additionally, it is recommended to use clean materials and expedite procedures.

For these reasons, protein purification method 2 is recommended for dsRBD3-End, dsRBD3-EndΔSSM, dsRBD4-End, and dsRBD4-EndΔSSM because these proteins are less prone to degradation and aggregation. Protein purification method 3 is suggested for hStau1 to prevent aggregation. Finally, protein purification method 4 for hStau1ΔSSM is the best choice to minimize protein degradation. These protocols were used to obtain pure, native and undegraded samples. Moreover, they can also be used for other proteins and can be easily modified to properly purify different proteins.

## Limitations

These methods were successfully used for Staufen purifications. These methods could also be applied to other basic and water-soluble proteins, including flexible polypeptides with disordered linkers. These methods can be modified according to the experimental needs of the researchers. These protocols cannot be used for the purification of liposoluble or acidic proteins.

## Troubleshooting

**Table:** 

Problem	Solution
Protein degradation	Use these protocols, EDTA and work fast, especially in the first steps of protein purification, when the samples are still in contact with high quantities of *E. coli* proteases.
Protein aggregation	This problem often occurs in TEV cleavage. In this case, do not let dialyze the protein for too long time. In general, no TEV cleavage should last >24 h. Add EDTA. Increase salt concentration or reduce protein concentration by adding buffer inside the dialysis bag. pI and pH values should not be very similar, otherwise hydrophobic interactions may prevail and may trigger protein degradation.
The protein sample fails to bind to the ion exchange column	Check buffer composition and pH. In case of a positive ion exchange column, buffer pH needs to be lower than pI. Check if the FPLC tubes are connected to the right buffers. The protein must have the proper charge to bind to these columns. Give the dialysis the time to work, dialysis steps shorter than 6 h can easily display this problem.
The protein does not bind RNA	Check sample quality on a SDS gel. Use cation exchange columns, a protein sample that does not bind to the column may be already bound to RNA molecules, in this case use the procedure described afterwards. It is also possible to use an anion exchange column, but in this case, it is necessary to use the flowthrough sample. Check buffer pH and pI.
The protein particles are already bound to RNA	In this case, run the sample into a negative ion exchange column. If dialysis was properly performed, the sample found in the flowthrough fractions should not contain RNA molecules.

## Ethics statements

This paper does not contain any data obtained from animals, humans or social media.

## CRediT author statement

Huma Shakoor and Andrea Tripepi performed the experiments described here, designed the experiments, evaluated the data, and wrote this article.

## Declaration of competing interest

The authors declare the following financial interests/personal relationships which may be considered as potential competing interests: the authors requested a patent for proteins with deleted or modified SSM. Patent application 102026000009622 submitted at the Italian Ministry of Enterprises and Made in Italy.

## Data Availability

Data will be made available on request.

## Appendix Supplementary materials

**Fig. 1 Supplementary Materials:** double Histidine tag and TEV cleavage site, hStau1, hStau1ΔSSM, dsRBD3-End, dsRBD3-EndΔSSM, dsRBD4-End, dsRBD4-EndΔSSM sequences. Histidine tag sequences are written in red and TEV cleavage site is highlighted in yellow. dsRBD2, dsRBD3, dsRBD4, TBD, SSM and dsRBD5 are represented in red, green, purple, grey, blue and gold respectively. Image, application 1

**Fig. 2 Supplementary Materials:** (A) protein marker pmVI; (B) SDS race of the cleanest GF fractions in 3, Δ3, 4, Δ4, Stau, ΔStau represent dsRBD3-End, dsRBD3-EndΔSSM, dsRBD4-End, dsRBD4-EndΔSSM, hStau1, hStau1ΔSSM, respectively; purified by protein purification method 1; (C) from left to right: degradation in hStau1ΔSSM in buf. A* after 12 h at 4 °C, and in TEV buffer after 0, 3, 5, 8 hours at 22 °C. Image, application 2
